# Author Correction: Metafiber transforming arbitrarily structured light

**DOI:** 10.1038/s41467-024-45398-6

**Published:** 2024-02-02

**Authors:** Chenhao Li, Torsten Wieduwilt, Fedja J. Wendisch, Andrés Márquez, Leonardo de S. Menezes, Stefan A. Maier, Markus A. Schmidt, Haoran Ren

**Affiliations:** 1 Chair in Hybrid Nanosystems, Nanoinstitute Munich, Faculty of Physics, Ludwig Maximilian University of Munich, 80539 Munich, Germany; 2 Leibniz Institute of Photonic Technology, 07745 Jena, Germany; 3 I.U. Física Aplicada a las Ciencias y las Tecnologías, Universidad de Alicante, P.O. Box 99, 03080 Alicante, Spain; 4 Dpto. de Física, Ing. de Sistemas y Teoría de la Señal, Universidad de Alicante, P.O. Box 99, 03080 Alicante, Spain; 5 Departamento de Física, Universidade Federal de Pernambuco, 50670-901 Recife-PE, Brazil; 6 School of Physics and Astronomy, Faculty of Science, Monash University, Melbourne, VIC 3800 Australia; 7 Department of Physics, Imperial College London, London, SW7 2AZ UK; 8 Abbe Center of Photonics and Faculty of Physics, FSU Jena, 07745 Jena, Germany; 9 Otto Schott Institute of Material Research, FSU Jena, 07745 Jena, Germany

**Keywords:** Metamaterials, Nanophotonics and plasmonics, Fibre optics and optical communications

Correction to: *Nature Communications* 10.1038/s41467-023-43068-7, published online 09 November 2023

The main manuscript was initially published with an error in the reference list, lacking page numbers. The authors have now submitted an updated reference list in a PDF/Word file, highlighting references with missing page numbers. Additionally, they propose replacing a previous reference (Ref. 56) with a more appropriate citation for the manuscript’s context.

Also, a minor error in Figure 5(e) was identified with the wrong placement of the red arrows – the authors have rectified this by providing an updated Figure 5, correcting the placement of red arrows.

