# Supplementary material for: Metafiber transforming arbitrarily structured light
Source: Nat Commun. 2023 Nov 9;14:7222. doi: 10.1038/s41467-023-43068-7 (PMC10632407; doi:10.1038/s41467-023-43068-7)
Supplement: Supplementary file 1 — Supplementary Information [file 41467_2023_43068_MOESM1_ESM.pdf]

Supplementary Information

Metafiber transforming arbitrarily structured light

Chenhao Li[1], Torsten Wieduwilt[2], Fedja J Wendisch[1], Andrés Márquez[3,4], Leonardo de S. Menezes[1,5], Stefan A. Maier[1,6,7,*], Markus A. Schmidt[2,8,9,*], Haoran Ren[6,*]

1. Chair in Hybrid Nanosystems, Nanoinstitute Munich, Faculty of Physics, Ludwig Maximilian University of Munich, 80539, Munich, Germany.
2. Leibniz Institute of Photonic Technology, 07745 Jena, Germany.
3. I.U. Física Aplicada a las Ciencias y las Tecnologías, Universidad de Alicante, P.O. Box 99, 03080 Alicante, Spain.
4. Dpto. de Física, Ing. de Sistemas y Teoría de la Señal, Universidad de Alicante, P.O. Box 99, 03080 Alicante, Spain.
5. Departamento de Física, Universidade Federal de Pernambuco, 50670-901 Recife-PE, Brazil.
6. School of Physics and Astronomy, Faculty of Science, Monash University, Melbourne, Victoria 3800, Australia.
7. Department of Physics, Imperial College London, London, SW7 2AZ, UK.
8. Abbe Center of Photonics and Faculty of Physics, FSU Jena, 07745 Jena, Germany.
9. Otto Schott Institute of Material Research, FSU Jena, 07745 Jena, Germany.

Emails: stefan.maier@monash.edu; markus.schmidt@leibniz-ipht.de; haoran.ren@monash.edu.

This supplementary material includes:

1. Supplementary Note 1. Characterization of the beam at the fiber end face.
2. Supplementary Note 2. The effect of the interference pattern created by a tower fiber on the imaging results.
3. Supplementary Note 3. Simulation of electric field intensity distribution inside nanopillar.
4. Supplementary Note 4. Jones matrix analysis based on a 3D meta-atom.
5. Supplementary Note 5. Height calibration of nanopillars.
6. Supplementary Note 6. Size calibration of 3D laser-nanoprinted nanopillars.
7. Supplementary Note 7. Conversion efficiency of a half-wave-plate metasurface sample.
8. Supplementary Note 8. Zoom in image of printed metasurface.
9. Supplementary Note 9. Set up of experiment for momentum-space imaging.
10. Supplementary Note 10. Intensity maps at different propagation distances in real space.
11. Supplementary Note 11. Impact of misalignment between metasurface and the fiber output.
12. Supplementary Note 12. Theoretical Fourier plane images of SLGM-5.

Supplementary Note 1. Characterization of the beam at the fiber end face

The characterization of the distribution of the light field at the end face of the fiber before the tower relies on characterizing the output beam of the blank fiber (PM-SMF, PM1550-XP, Thorlabs). In detail, we measured the beam diameter ($\lambda_0 = 1550\ nm$) for different distances from the fiber surface and compared it with the theory of Gaussian beams (Fig. S1), allowing us to determine the size of the beam at the fiber surface[1].

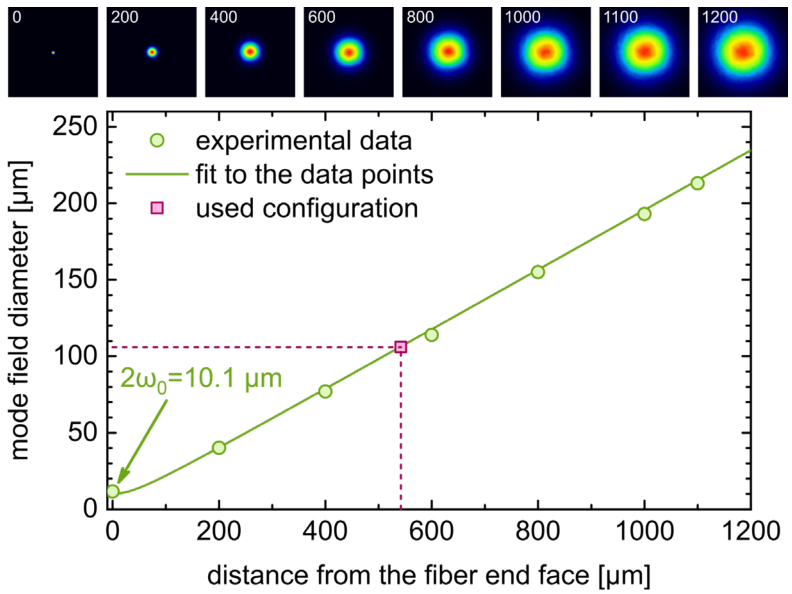

Fig. S1. Characterization of the output beam of the fiber used (PM-SMF, PM1550-XP, Thorlabs) without the tower. The points refer to the measured mode field diameter at selected distances from the fiber end face, while the line is a fit to the data points. The top images refer to the measured beam patterns, with the respective numbers in the top left corners referring to the distance to the fiber surface (in µm). The red square indicates the height of the tower used in the metasurface-based experiments discussed in the manuscript.

Within the context of Gaussian beams, the beam radius is defined with respect to the transverse position at which the intensity has dropped to $1/e^2$, is given by

$$w(z) = w_0\sqrt{1 + (z/z_R)^2},$$

with the beam waist w_0, the longitudinal position z and the Rayleigh length z_R. The latter is defined by $z_R = \pi w_0^2/\lambda_0$ (vacuum wavelength $\boldsymbol{\lambda_0}$). By fitting the measured data (points in Fig. S1) with the equation stated above, a beam diameter at the fiber surface of $w_0^{exp} = 10.1\ \mu m$ is obtained, which fits well with the value from the datasheet $w_0^{data} = 10.1\ \mu m$. It is important to mention that the measurements show field patterns with circular distributions at any distance from the fiber end face (top images in Fig. S1), which is important to consider in case metastructures are created on the tower.

Supplementary Note 2. The effect of the interference pattern created by a tower fiber on the imaging results

The tower was designed to be hollow to reduce its impact on the output light, although we found that the inner diameter of the tower (90 μm) is smaller than the beam at the top part of the tower. As a result, some part of the beam is reflected and causes a ring-shaped interference pattern on the top of the tower, which might influence the wavefront shaping process (Fig. S2b). We simulated the interference of such a ring-shaped interference pattern on the imaging results of both the polarization and phase singularity beams in the Fourier plane. Our simulation shows that the ring-shaped interference is related to high spatial-frequency components in the Fourier plane and does not change the central part of the polarization and phase singularity beams in our imaging results (Figs. S2d and S2f).

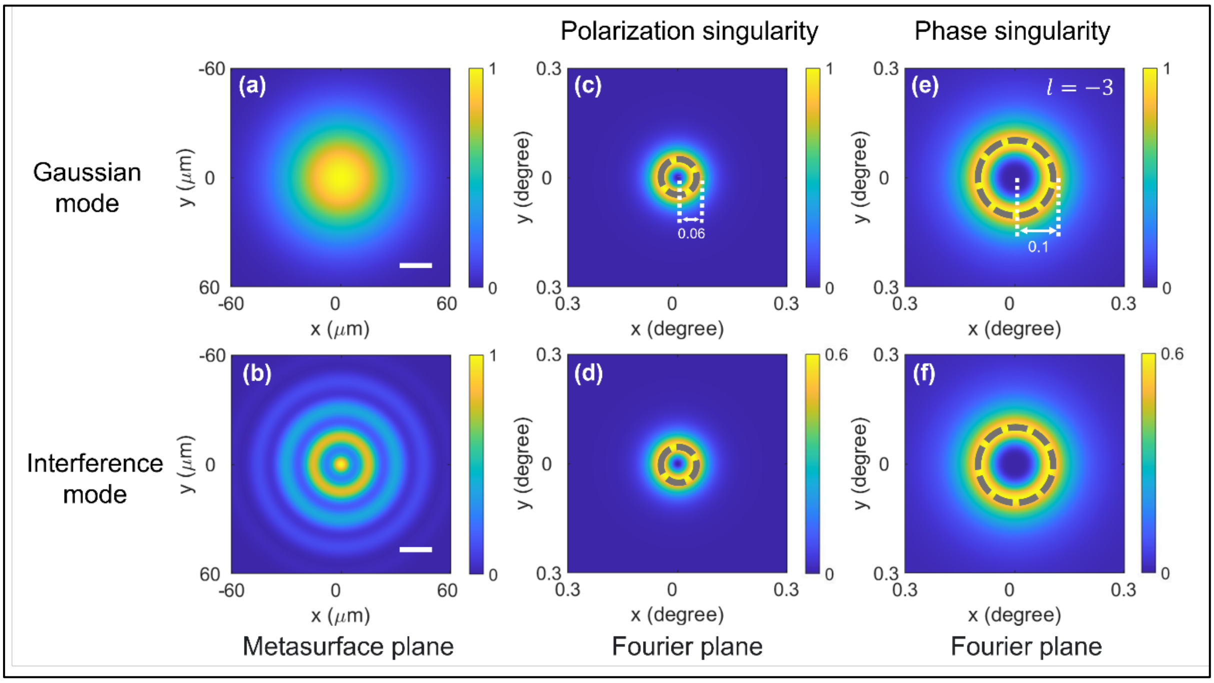

Fig. S2. Simulation of influence of the ring-shaped interference pattern induced by a tower on the imaging results of both the polarization and phase singularity beams. (a and b) Intensity distributions of a Gaussian mode and a ring-shaped interference mode (due to a fiber tower reflection) at the metasurface plane, respectively (scale bar: 25 μm). (c and d) Intensity distributions of a polarization singularity beam (azimuthally polarized beam) in the Fourier plane under the illumination of a Gaussian mode (c) and a ring-shaped interference mode (d), respectively (e and f). The counterparts of (c and d) but with an OAM-carrying phase singularity beam of l=3. The dashed circles mark the beam divergence angle of 0.06 (c and d) and 0.1 (e and f) respectively.

In our experiment, we can further optimize our design of the hollow tower by including an extended upper region for larger distances from the fiber end face (Fig. S3). This allows for the inner hollow part of the tower to gradually increase up to a diameter of 130 μm at the top, preserving the Gaussian mode profile of the fiber output. However, the new tower results are preliminary and require much more work to enhance its mechanical robustness for hosting metasurfaces, and therefore, we would opt not to use them in current work.

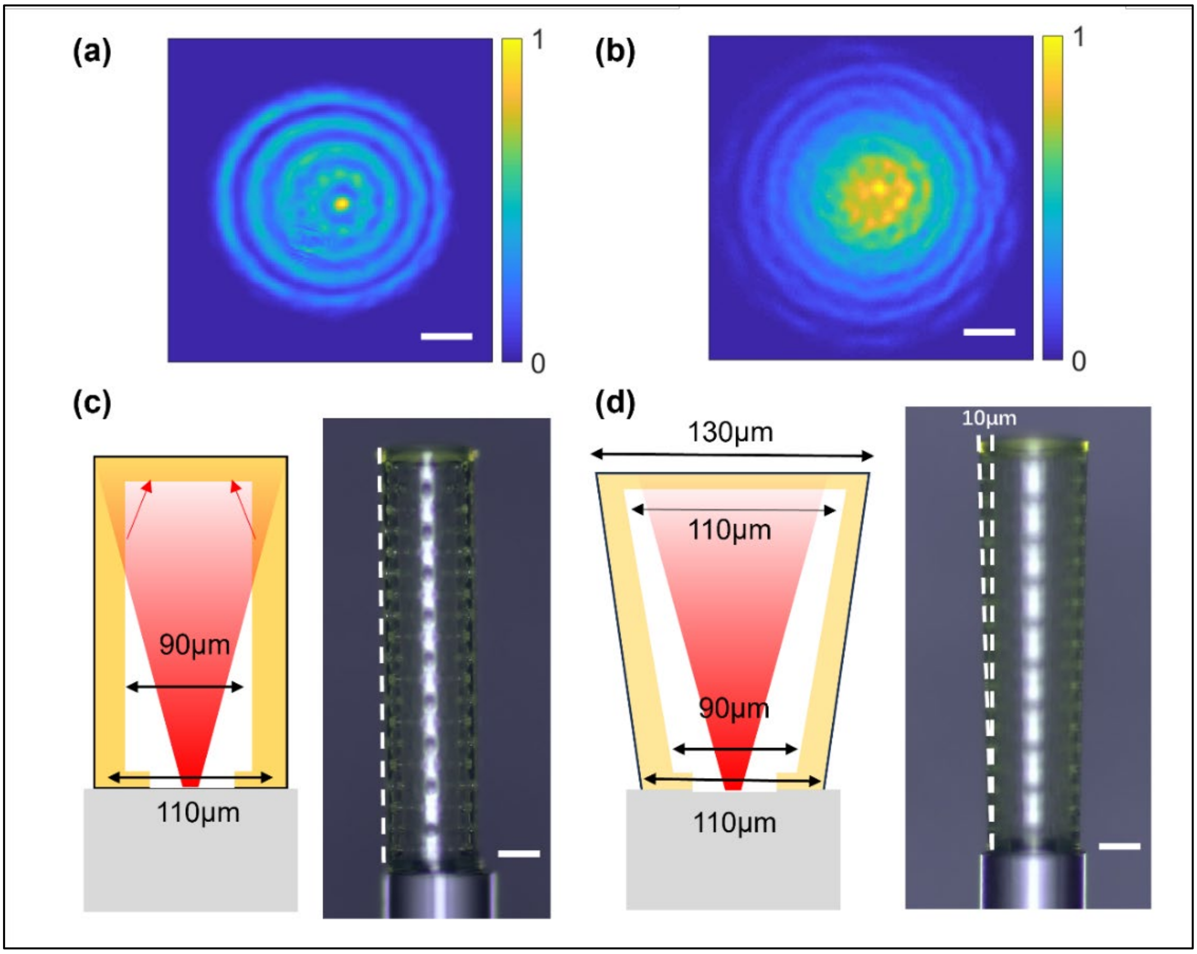

Fig. S3. Intensity distributions of fiber outputs on the surface of hollow tower structures printed on the end faces of SMF-28 fibers. (a) The output intensity distribution of a straight hollow tower with a diameter of 90 μm. (b) The output intensity distribution of an expanded hollow tower that leads to an extended upper part with a diameter of 130 μm. Scale bar: 20 μm (c and d) Schematics and microscope images of the straight tower (c) and the enlarged tower (d), respectively. Scale bars: 50 μm.

Supplementary Note 3. Simulation of electric field intensity distribution inside nanopillar

To verify that the Jones formalism can be used in this work, finite element simulations have been employed to calculate the two orthogonal modes in the 2D cross-sections of the meta-toms (Fig. S4, $n_{polymer}$ = 1.5, n_{air} = 1, W = 550 nm, L = 1.6 μm, P = 2.2 μm, λ = 1550 nm). The results clearly show that the orientation of the electric field vectors is along either the x' or y' direction for virtually all points within the cross-section of the meta-atom, corresponding to a linear polarization for nearly the entire field. As expected, only very minor deviations are seen at the corners of the meta-atom, which are of no consequence and can be neglected. This minor deviation is expected and can be explained from a waveguide perspective by the small refractive index difference between core (polymer) and cladding (air). It should be noted that for larger refractive index differences (e.g. silicon to air), a stronger deviation from linear behavior can be expected. Overall, it can be stated that the modes in the polymeric meta-atoms used here are completely linearly polarized, allowing the use of the Jones vector formalism.

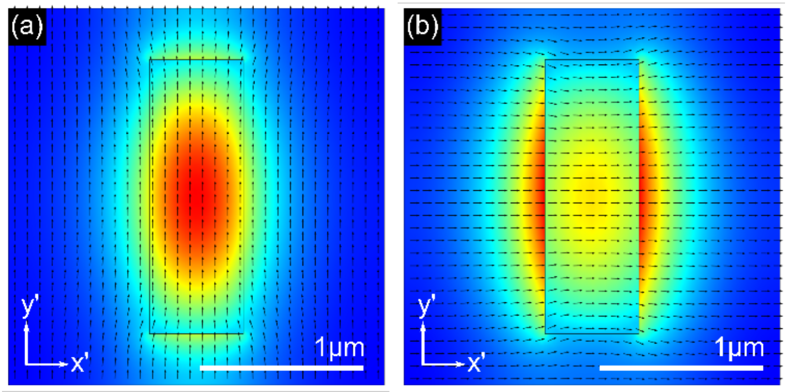

Fig. S4: Spatial distribution of the intensity (linear scale) and the orientation of the in-plane electric field (black arrows with normalized length) of the two relevant modes within the meta-atoms, simulated using finite element modelling. The left plot (a) shows the mode with a dominant polarization along the y'-direction, while the right plot (b) shows the orthogonal mode. The coordinate system (x'y'-system) shown in the bottom left corners of the two plots refers to the orientation of the cross-section of the meta-atom and not to the laboratory system.

Supplementary Note 4. Jones matrix analysis based on a 3D meta-atom

The metasurface unit cell can be described by the Jones matrix of a linearly birefringent wave plate:

$$\mathrm{M} = R(\gamma)\begin{bmatrix} t_x e^{i\varphi_\mathrm{x}} & 0 \\ 0 & t_y e^{i\varphi_y} \end{bmatrix} R^{-1}(\gamma)$$

Here, the element imposes both amplitude (t_x, t_y) and phase (φ_x, φ_y) modulations on light linearly polarized along the x and y axes. $R(\gamma)$ is the rotation matrix $\begin{bmatrix} \cos(\gamma) & \sin(\gamma) \\ -\sin(\gamma) & \cos(\gamma) \end{bmatrix}$, which represents a coordinate system transformation. γ is the in-plane rotation angle of the nanopillar in the counterclockwise direction starting from the x axis.

Thus, for an arbitrary input light with characteristic electric field E_{in} passing through the meta-atoms, the output light electric field can be described by $E_{out} = ME_{in}$.

In this context, our incident light is linear polarized along the x axis with a Jones vector given by $\begin{bmatrix} 1 \\ 0 \end{bmatrix}$. So, the output light electric field is expressed as:

$$E_\mathrm{out} = \begin{bmatrix} t_\mathrm{x}\cos^2(\gamma)e^{i\varphi_\mathrm{x}} + t_\mathrm{y}\sin^2(\gamma)e^{i\varphi_y} \\ \cos(\gamma)\sin(\gamma)\,t_\mathrm{y}e^{i\varphi_y} - \cos(\gamma)\sin(\gamma)\,t_\mathrm{x}e^{i\varphi_x} \end{bmatrix}$$

If we make some simplifications, we get to:

$$E_\mathrm{out} = e^{i\varphi_\mathrm{x}}\begin{bmatrix} t_\mathrm{x}\cos^2(\gamma) + t_\mathrm{y}\sin^2(\gamma)e^{i\Delta\varphi} \\ \frac{1}{2}(t_\mathrm{x} - t_\mathrm{y}e^{i\Delta\varphi})\sin(2\gamma) \end{bmatrix}$$

Where $\Delta\varphi = \varphi_\mathrm{x} - \varphi_\mathrm{y}$ is the phase difference between the modes polarized along the x and y axes, respectively.

Supplementary Note 5. Height calibration of nanopillars

To calibrate the height of nanopillars, we fabricated nanopillars with height variation from 6 μm to 10 μm with an increment of 1 μm, which cover most heights defined in our 3D design library. To make the measurement more accurate, we decided to push down the nanopillars instead of measuring height at a tilt angle of SEM imaging. It should be mentioned that the height resolution (smallest variation in height) is controlled by the slicing accuracy in the nanoprinting process, which was set to be 20 nm in our experiment. We used this small slicing distance (much smaller than its typical use) to enhance the mechanical strength of high-aspect-ratio nanopillars to avoid collapsing. To provide a precise characterization, the actual procedure includes fabrication of nanopillars of different heights on a glass substrate, turning the sample upside down and placing it on a flat surface, and slightly sliding the sample. This forces the nanopillars to be pushed down on the surface. Undamaged pillars were measured under SEM and compared to the designed heights. Thus, we obtain a relationship between design and the actual height of the nanopillars. Selected SEM images of nanopillars and their corresponding heights are shown in Fig S5.

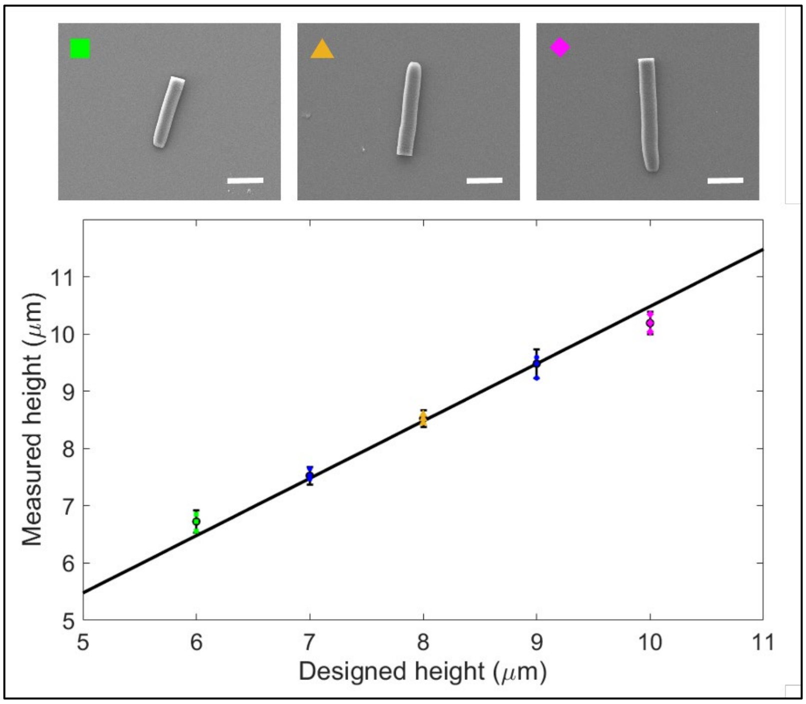

Fig. S5. Height calibration of nanopillars. Top images: SEM images of three selected nanopillars with designed heights of 6 μm, 8 μm and 10 μm, respectively. (Scale bars: 3 μm). The black solid line is the fitted relationship between designed height and the measured height.

The relationship between designed height and measured height is linearly fitted with the following expression, with an offset arising from the axial extension of the focal spot:

$$H_{measured} = H_{design} + 0.48\ \mu\text{m}$$

Supplementary Note 6. Size calibration of 3D laser-nanoprinted nanopillars

Due to the photopolymerization process, the fabricated structures are generally different from the designed ones. To make the metasurface more accurate, we first calibrated the geometric size of nanopillars. Here we fabricated a set of identical nanopillars with different sizes and tested the repeatability and we found that the fabrication uncertainty (due to machine error) in the lateral directions is as small as 15 nm.

In lateral size calibration, the calibration was done for nanopillar length and width dimensions. In horizontal direction, we design different nanopillars with length changing from 600 nm to 1200 nm with a step of 100 nm. We measured the length of pillars under SEM and obtained the relationship between the design and measured results by fitting (Fig S6).

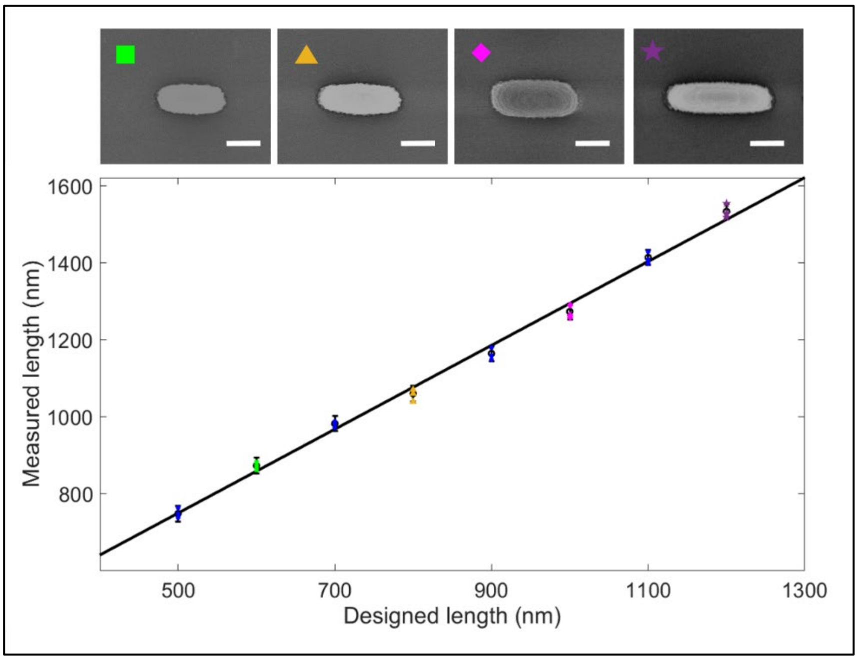

Fig. S6. Calibration of the length size of 3D laser-nanoprinted nanopillars. The images on top are the SEM image of nanopillars with different lengths (scale bar: 500 nm). Left to right: nanopillars with designed lengths of 600 nm, 800 nm, 1000 nm and 1200 nm. The black solid line is the fitted relationship between designed length and the measured length.

There is a linear relationship between the design and the actual printing size. So, the fitting relationship is:

$$L_{measured} = 1.09 * L_{designed} + 205.2 \text{ nm}$$

The same method is used for the width characterization and the experiment results are shown in Fig S7. Similarly, the relationship between design and actual printed size can be written as:

$$W_{measured} = 1.03 * W_{designed} + 341.2 \text{ nm}$$

The offset values in the lateral directions are determined from the diffraction-limited focal spot of a tightly focused linearly polarized light.

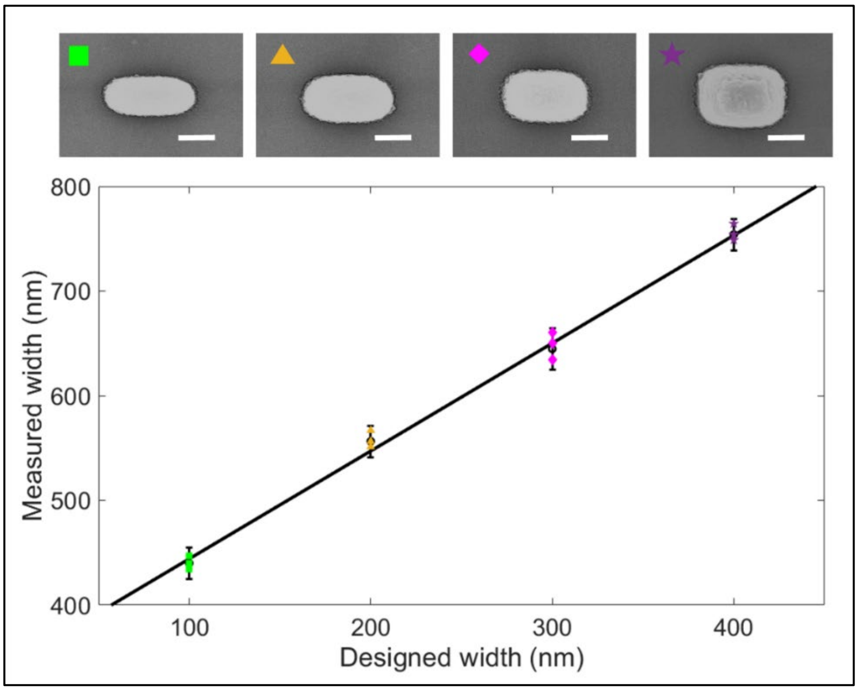

Fig. S7. Calibration of the width size of 3D laser-nanoprinted nanopillars. The images on top are the SEM image of nanopillars with different widths (scale bar: 500 nm). Left to right: nanopillars with designed widths of 100 nm, 200 nm, 300 nm and 400 nm. The black solid line is the fitted relationship between designed width and the measured width.

Supplementary Note 7. Conversion efficiency of a half-wave-plate metasurface sample

Our metasurface consists of many subwavelength pixels that can be regarded as waveplates converting incident linear polarization into spatially variant polarization states on a vector beam. Since we have demonstrated many different polarization outputs including linear, circular, and elliptical polarizations, to simplify the verification process, here fabricated a half wave-plate-type metasurface on glass and measured its conversion efficiency of 67%. This represents the case that our metasurface can convert the left-handed circularly polarization (LCP) into right-handed circularly polarization (RCP), and vice versa. Our measurement setup is shown in Fig S8. The conversion efficiency is defined as the intensity ratio of the cross-polarization component normalized to the incident light, e.g., IRCP/ILCP.

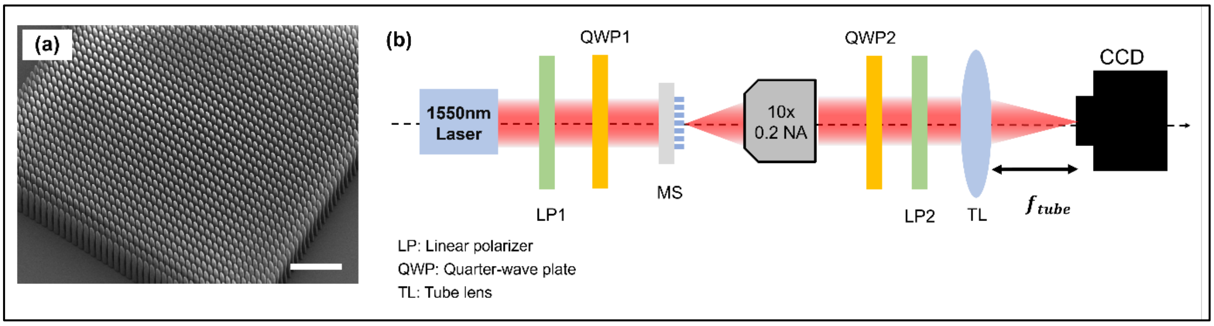

Fig. S8. (a) SEM image of a half-wave-plate-type nanopillar metasurface (scale bar: 20 μm). (b) Optical setup for measuring polarization conversion efficiency of the metasurface on a glass substrate.

Supplementary Note 8. Zoom in image of printed metasurface

To reveal the morphology of the metasurface and the tower more clearly, we have provided more SEM images of SLGM-1 in Fig. 3d (Fig. S9).

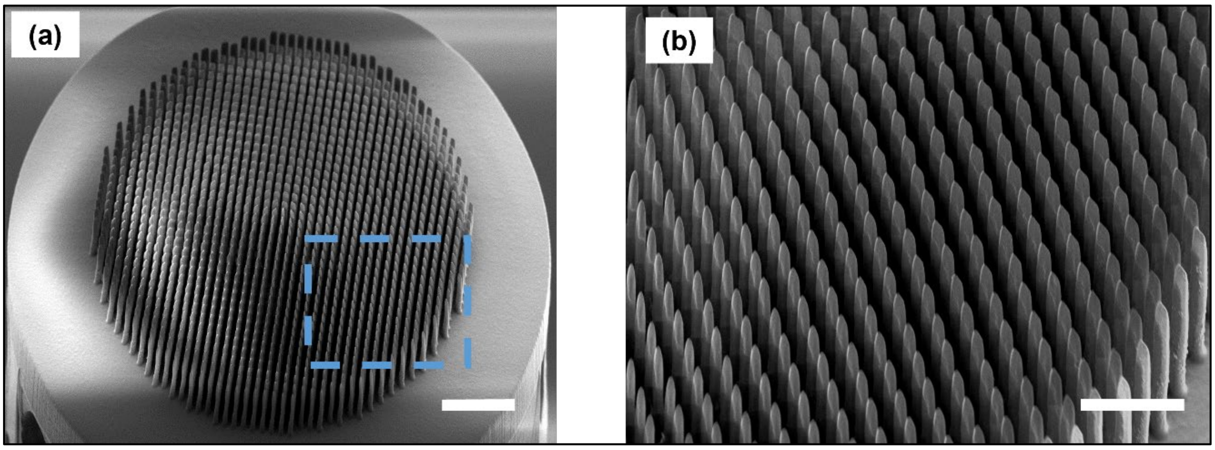

Fig. S9. Example SEM images of SLGM-1 used for creating the radial vector beam. (a) SEM image of the whole metasurface (scale bar: 20 μm). (b) Zoom-in area of (a) (scale bar: 8 μm).

Supplementary Note 9. Setup of experiment for momentum-space imaging

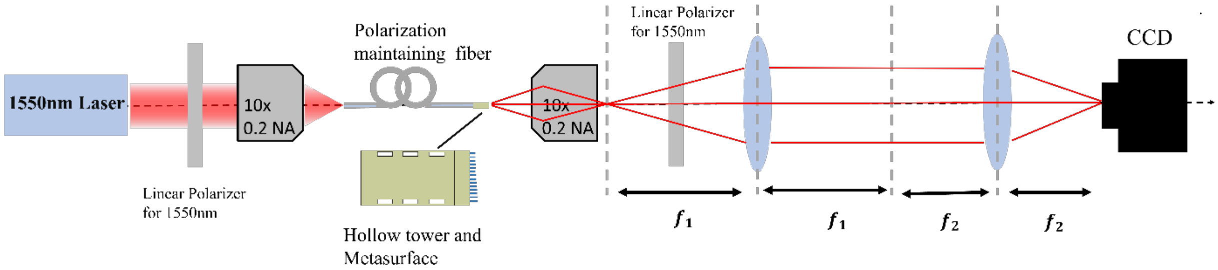

Fig. S10: Optical setup for SLM characterisation in momentum-space. The laser source is a supercontinuum laser source (SuperK Fianium, NKT Photonics) and selected by wavelength selector (SuperK Select, NKT Photonics) with a wavelength at 1550 nm. The beam passed a linear polarizer and coupled into a polarization maintaining fiber with a 10x objective (Olympus). A $4f$ system (f_1 is 300mm and f_2 is 200 mm) was used to characterize the back focal plane image and the results were recorded with a near-infrared camera (Raptor, Owl 640 M).

Supplementary Note 10. Intensity maps at different propagation distances in real space

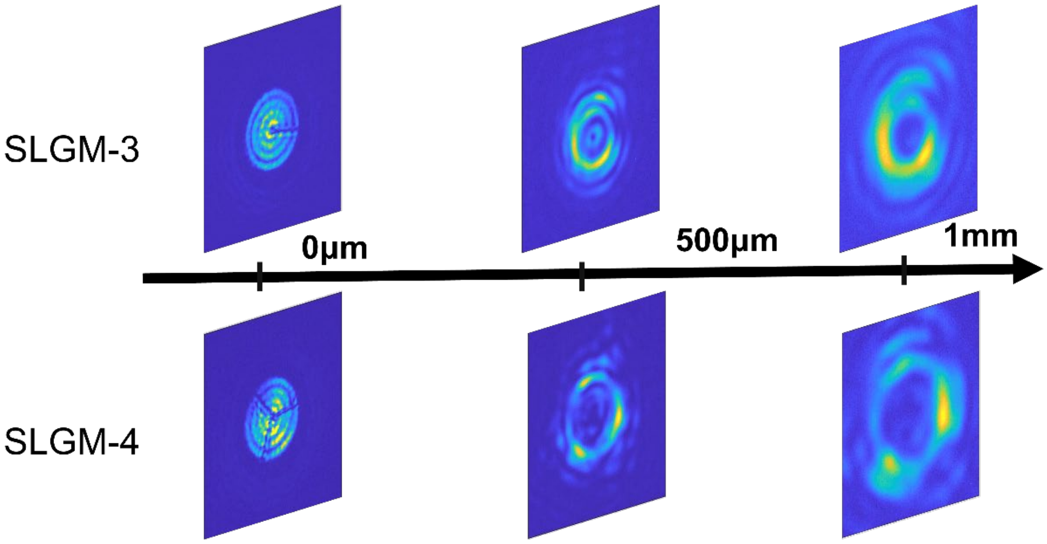

Fig. S11. Intensity distributions at different propagation distances from metasurface in real space for SLGM-3 (S=-1) and SLGM-4 (S=-3).

Supplementary Note 11. Impact of misalignment between metasurface and the fiber output.

According to our simulations, we attribute the main reason for the deviation is due to the misalignment of the fiber output and the metasurface at the exit plane of a fiber tower. A tilt of the fiber end face with respect to the fiber axis leads to a slightly tilted beam (Fig. S12a), which could be caused by imperfect fiber cleaving technique. This misalignment mainly results from the fiber cleaving quality, with the ideal situation that the cutting cross-section is flat and perpendicular to the fiber axis. For practical fiber cleaving, however, there is some imperfection that leads to a shifted fiber beam output. To verify its impact, we simulated the influence of misalignment for the case of the sample producing azimuthal output polarization. Here we assume that the metasurface was misaligned by 20 μm in x-direction and 5 μm along the y-axis (after propagating 550 μm from an imperfect cleaved fiber end face) with respect to the fiber output on the exit plane of the fiber tower. Here we calculate the impact of this misalignment on the generated OAM modes with different topological charges of -1 and -3 (Fig. S12).

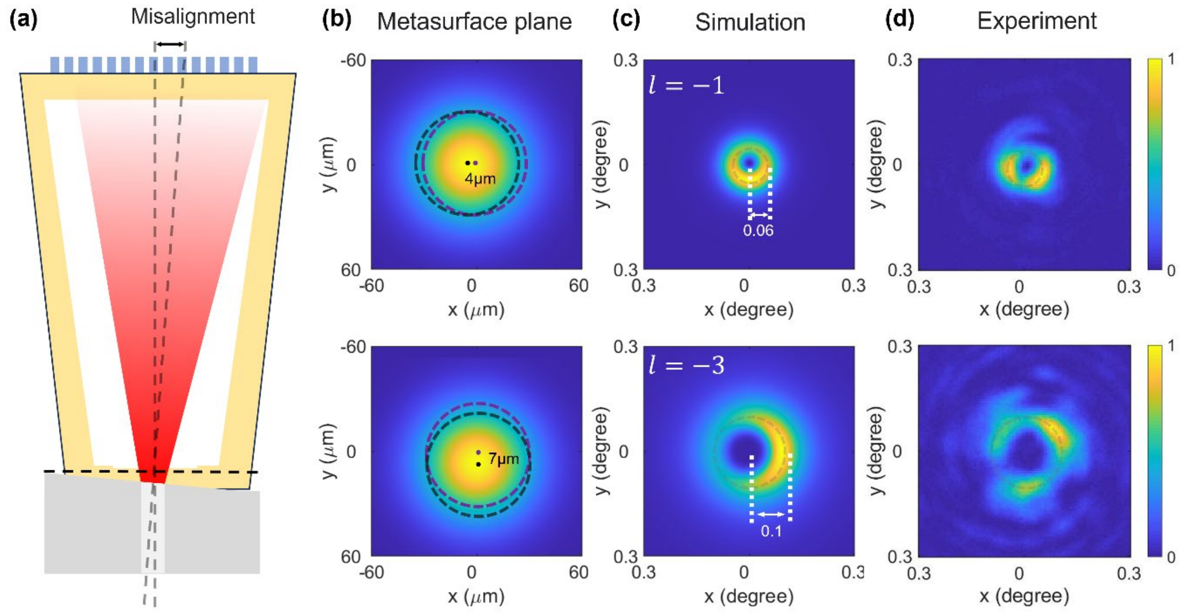

Fig. S12. Impact of misalignment between the fiber output and metasurface on the structured light transformation. (a) Schematic of misalignment resulting from the cleaved fiber end face not being perpendicular to the fiber axis. (b) Simulation results of the OAM beam output carrying *l*=-1, considering a 4 μm shift along the x-direction (top), and output beam carrying *l*=-3 with a 7 μm shift along the y-axis in the metasurface plane (bottom). The black and purple dots and the corresponding dotted lines represent the beam and metasurface, respectively. (c) Simulation results in Fourier Plane with the incident beam in (b). (d) The experiment results in our SLGM-3 and 4. The dashed circles in (c) mark the beam divergence angle of 0.06 and 0.1, respectively.

Supplementary Note 12. Theoretical Fourier plane images of the SLGM-5.

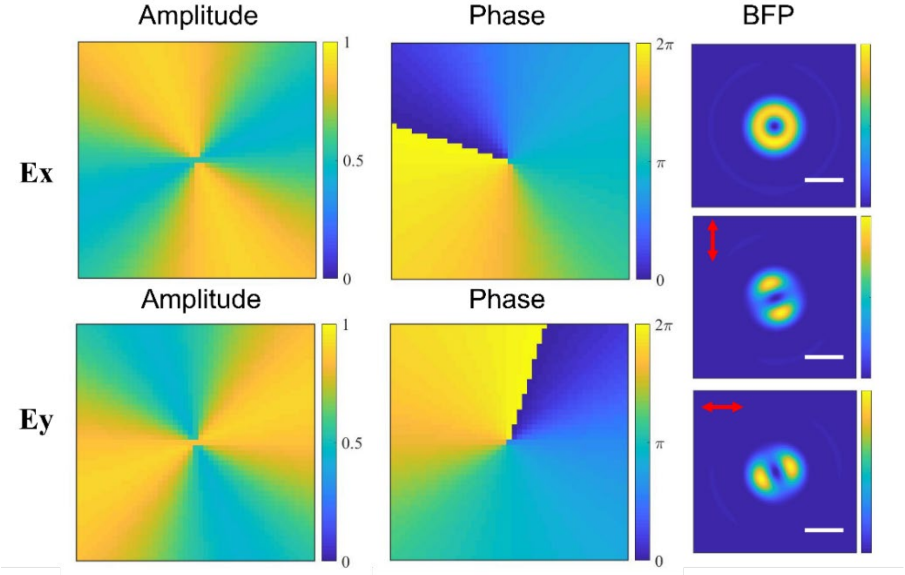

Fig. S13. Amplitude and phase distributions of the E_x and E_y polarizations used for implementing the SLGM-5. Theoretical intensity distributions of the SLGM-5 fibre in the Fourier plane are shown in the right panel, where red arrows mark the polarization filtering angles. Scale bars on the right panel represent 0.1 degrees.

Supplementary References

[1]. B. E. A. Saleh, M. C. Teich, Fundamentals of Photonics (1991).
